# [Retracted] Grooved hydroxyapatite scaffold modulates mitochondria homeostasis and thus promotes osteogenesis in bone mesenchymal stromal cells

**DOI:** 10.3892/mmr.2025.13519

**Published:** 2025-04-07

**Authors:** Chenglong Li, Lu Yang, Xiaohua Ren, Mu Lin, Daonan Shen, You Li, Xiangyu Zhang, Chunhui Liu, Yandong Mu

Mol Med Rep 22: 2801–2809, 2020; DOI: 10.3892/mmr.2020.11352

Following the publication of the above paper, it was drawn to the Editor's attention by a concerned reader that certain of the fluorescence microscopy images shown in Fig. 2C on p. 2805 were strikingly similar to data that had appeared previously in other papers written by different authors at different research institutes.

In view of the fact that the abovementioned data had already apparently been published prior to its submission to *Molecular Medicine Reports*, the Editor has decided that this paper should be retracted from the Journal. The authors were asked for an explanation to account for these concerns, but the Editorial Office did not receive a satisfactory reply. The Editor apologizes to the readership for any inconvenience caused.

